# Legalization of cannabis for medical and recreational use

**DOI:** 10.1192/j.eurpsy.2023.83

**Published:** 2023-07-19

**Authors:** D. Hasin

**Affiliations:** ^1^ New York State Psychiatric Institute; ^2^Department of Epidemiology, Columbia University Mailman School of Public Health; ^3^Department of Psychiatry, Columbia University Irving Medical Center, New York, United States

## Abstract

**Abstract:**

Since 1996, 39 of the 50 US states have enacted medical cannabis laws (MCL) and since 2012, 21 states and Washington D.C. (DC) enacted recreational cannabis laws (RCL). Many individuals can use cannabis without harm, and legalization helps achieve social justice and financial aims. However, 20%-33% of cannabis users develop cannabis use disorder (CUD), which is associated with impaired functioning, psychosocial, physical and psychiatric problems. Despite these risks, Americans increasingly see cannabis use as harmless or even beneficial in treating or preventing health problems. The prevalence of frequent cannabis use and CUD has increased in US adults in recent years. Studying the role of MCL and RCL in these nationally increasing prevalences is challenging due to staggered-adoption dates of state legalizations, few years of data available to study RCL, and other potential influences on cannabis use and CUD. Using self-report data from US national surveys, MCL have been shown to have little influence on adolescent cannabis use, but increase adult illicit cannabis use and CUD. Fewer studies have examined RCL; in these, RCL increases adult use and CUD. However, studies are needed in national patient populations with multiple risk factors for CUD, including painful medical conditions and a high prevalence of psychiatric disorders. We used data from the electronic health records (EHR) database of the US Veterans Health Administration (VHA), the largest integrated healthcare system in the US, to examine trends in provider-diagnosed ICD-9-CM and ICD-10-CM CUD over time, differences in these trends by patient characteristics, and the role of MCL and RCL in the trends. CUD diagnoses more than doubled overall in the VHA, from 0.85% in 2005 to 1.92% in 2019. Increases were found across age, sex, and racial/ethnic subgroups of patients, with greater rates and increases among patients with chronic pain and with psychiatric disorders. Among patients living in MCL and RCL states, increases in CUD were larger than among patients in other states, although the size of legalization effects suggested that other factors are important in driving up prevalence, e.g., online commercialized information and other forms of advertising. The tensions between public health aims, social justice and financial gain will be discussed.

**Disclosure of Interest:**

None Declared

